# Limitations and perceived delays for diagnosis and staging of lung cancer in Portugal: A nationwide survey analysis

**DOI:** 10.1371/journal.pone.0252529

**Published:** 2021-06-04

**Authors:** Fernando Barata, Paula Fidalgo, Sara Figueiredo, Fernanda S. Tonin, Filipa Duarte-Ramos

**Affiliations:** 1 Centro Hospitalar e Universitário de Coimbra, Coimbra, Portugal; 2 Centro Hospitalar do Porto, Porto, Portugal; 3 Oncology-Medical Department, AstraZeneca Portugal, Portugal; 4 Pharmaceutical Sciences Postgraduate Program, Federal University of Paraná, Curitiba, Brazil; 5 Department of Social Pharmacy, Faculty of Pharmacy, University of Lisbon, Lisbon, Portugal; 6 EPIUnit—Instituto de Saúde Pública, University of Porto, Porto, Portugal; Chang Gung Memorial Hospital and Chang Gung University, Taoyuan, Taiwan, TAIWAN

## Abstract

**Background:**

We aimed to identify the perception of physicians on the limitations and delays for diagnosing, staging and treatment of lung cancer in Portugal.

**Methods:**

Portuguese physicians were invited to participate an electronic survey (Feb-Apr-2020). Descriptive statistical analyses were performed, with categorical variables reported as absolute and relative frequencies, and continuous variables with non-normal distribution as median and interquartile range (IQR). The association between categorical variables was assessed through Pearson’s chi-square test. Mann-Whitney test was used to compare categorical and continuous variables (Stata v.15.0).

**Results:**

Sixty-one physicians participated in the study (45 pulmonologists, 16 oncologists), with n = 26 exclusively assisting lung cancer patients. Most experts work in public hospitals (90.16%) in Lisbon (36.07%). During the last semester of 2019, responders performed a median of 85 (IQR 55–140) diagnoses of lung cancer. Factors preventing faster referral to the specialty included poor articulation between services (60.0%) and patients low economic/cultural level (44.26%). Obtaining National Drugs Authority authorization was one of the main reasons (75.41%) for delaying the begin of treatment. The cumulative lag-time from patients’ admission until treatment ranged from 42–61 days. Experts believe that the time to diagnosis could be optimized in around 11.05 days [IQR 9.61–12.50]. Most physicians (88.52%) started treatment before biomarkers results motivated by performance status deterioration (65.57%) or high tumor burden (52.46%). Clinicians exclusively assisting lung cancer cases reported fewer delays for obtaining authorization for biomarkers analysis (p = 0.023). Higher waiting times for surgery (p = 0.001), radiotherapy (p = 0.004), immunotherapy (p = 0.003) were reported by professionals from public hospitals.

**Conclusions:**

Physicians believe that is possible to reduce delays in all stages of lung cancer diagnosis with further efforts from multidisciplinary teams and hospital administration.

## Introduction

Lung cancer is the leading cause of cancer deaths worldwide, with one reported death every 18 seconds [1, 2]. In Portugal, this disease is the fourth most frequent type of tumor (9.1% of total cancers in 2018), with more than 4,500 reported deaths annually, which represents 16.1% of the mortality due to cancer in the country. Additionally, lung cancer is responsible for more than 5,000 hospitalizations every year, which represents an economic burden of one third of total costs with patients care [3, 4].

Previous studies have identified some factors associated to poor outcomes in lung cancer patients whose rates of 5-years overall survival usually range from 10% in Europe to around 15–18% in the United States [3]. These factors include, among others, social and economic inequalities, tobacco exposure, disease at more advanced stages, delayed referrals and diagnosis, and poor access to healthcare services and cancer care. Missed opportunities for earlier detection lead to increased patient distress, under-utilization of definitive therapy and potentially increased risk of death [5, 6].

Most cases of lung cancer (around 60–70%) are diagnosed in symptomatic patients that are usually at an advanced stage of the disease, which means a poorer prognosis and limited treatment [7]. If the diagnosis is performed in early stages of the disease, allowing for more immediate treatments, survival with curative-intent surgery rates can vary between 60% to 80% [2, 8]. In this scenario, the American Cancer Society recommends annual screening with low-dose computer tomography for certain people at higher risk for the disease (e.g. aged 55–74, currently smokers or with at least 30-pack-year smoking history) [9, 10]. In Europe, national health policy groups are discussing the implementation of screening grounded on evidence from randomized trials showing significant reduction of lung cancer mortality of 20–26% [11, 12]. However, controversies on lung cancer screening still exist, with special concerns on imaging workflow, radiation dose, management of small nodules, overdiagnosis bias, lead-time and length-time bias and cost-effectiveness of the procedure [13, 14].

Delays in diagnosis are one of the main factors associated with reduced survival rates. Clinical guidelines have been implemented in some countries to standardize the diagnosis process, better define the time from diagnosis to beginning of treatment, and to improve clinical results in lung cancer. The British National Health Service recommends a maximum waiting time of two-weeks (14 days) from primary health care referrals to the first consultation with a lung cancer specialist [15]. Treatments should be started within 31 days from the date of the clinical decision and up to 62 days from the date of the general practitioner’s referral. In the United States, it is recommended that patients wait no more than 10 days for consultation with a specialist and start treatment within 42 days of diagnosis. In Australia, periods of 14 days from the initial referral of the general practitioner to the first specialty consultation, and also from diagnosis to the first treatment are recommended [16]. However, previous scoping reviews showed an average time of 27 days for lung cancer diagnosis, with time distributions below those recommended by international guidelines (ranging from 6 to 45 days) [5, 17].

To date, few reports have assessed physicians’ perceptions of the current difficulties regarding the referral process of lung cancer patients, as well as the barriers for diagnosis and treatment [18–20], none of them performed in Portugal. Thus, we aimed to identify by means of a web-based nationwide survey, the perception among specialized physicians on the main limitations to a rapid diagnosis, staging and beginning of treatment in lung cancer that can impact on patient’s survival.

## Material and methods

### Study design and variables

We conducted a descriptive cross-sectional, 50-item, web-based survey (Google form) with convenience sampling between February 2020 and April 2020. Subjects were pulmonologists or oncologists in Portugal, specialists in lung cancer treatment, invited to participate the study via email. Participants were fully informed regarding the nature of the study, the procedures for data recording and the voluntary nature of their participation. Responders provided their electronic informed consent before survey’s completion, and anonymity was guaranteed. Only participants providing informed consent were included in the study and had access to the questionnaire. Participants’ withdrawal was allowed at any time. This study was waived of bioethical approval because it does not contain any intervention on human subjects nor individual health data (National legislation—Law 21/2014).

Survey questions were related to diagnosis and treatment patterns performed during the last semester 2019 and organized into the following sections: sociodemographic data (e.g. age, medical specialty, working place), patients’ referral process, histopathological diagnosis, disease stating, multidisciplinary consultations, biomarkers measurement and therapeutic approaches. The questionnaire was specifically developed for this study and reviewed by experts in the field. Participants took an average time of 10 min to complete the survey. Procedures followed standards for scientific research and were performed according to the Declaration of Helsinki.

### Data analysis

For analysis purposes, variables reassignment’s were performed, namely: physicians that reported to have more than 75% proportion of their patients diagnosed with lung cancer were considered to “exclusively assist patients with lung cancer”; according to healthcare setting, physicians were classified as working in public or private hospitals.

Variables’ normality was assessed with Kolmogorov-Smirnov and Shapiro-Wilk tests with additional visual inspection of the Q-Q plots. Descriptive statistics were used to summarize the data, with absolute and relative frequencies to describe categorical variables and the median, interquartile range (IQR), and minimum and maximum values for continuous (non-normal) variables. The association between categorical variables was assessed through Pearson’s chi-square test, while the Mann-Whitney U test was used to compare differences between two independent groups when the dependent variable was continuous, but not normally distributed. Results were reported with 95% confidence intervals (CI). Analyses were conducted in Stata Statistical Software version 15.0 SE (College Station, TX: StataCorp LL) and p-values below 5% were considered statistically significant.

## Results

Overall, 91 physicians from all the national hospitals that have lung cancer treatment departments in Portugal were invited to participate the study. The final panel (e.g. physicians that completely responded the survey) is represented by 61 physicians (response rate of 67.03%). Tables 1 and 2 show the sociodemographic characteristics of the participants and their perception on the limitations in the diagnosis and staging of lung cancer in Portugal.

**Table 1:** Baseline characteristics of 61 physicians in Portugal.

Variables/categories	Total n (%)
**Clinical specialty**	
Oncology	16 (26.23%)
Pulmonology	45 (73.77%)
**Region**	
North	21 (34.43%)
Center	10 (16.39%)
Lisbon e Vale do Tejo	22 (36.06%)
Alentejo	2 (3.28%)
Algarve	3 (4.92%)
AR–Madeira	1 (1.64%)
AR–Açores	2 (3.28%)
**Healthcare setting**	
Public hospital	55 (90.16%)
Private hospital	6 (9.84%)
**Referral process**	
**Primary care**	
<25% patients	29 (47.54%)
25–50% patients	13 (21.31%)
50–75% patients	7 (11.47%)
>75% patients	1 (1.64%)
N/A	11 (18.03%)
**Urgency department**	
<25% patients	25 (40.98%)
25–50% patients	12 (19.67%)
50–75% patients	7 (11.47%)
>75% patients	3 (4.92%)
N/A	14 (22.95%)
**Others**	
<25% patients	26 (42.62%)
25–50% patients	6 (9.84%)
50–75% patients	7 (11.47%)
>75% patients	6 (9.84%)
N/A	16 (26.23%)

Note: AR: autonomous region; N/A: not answered

**Table 2:** Perceived barriers to diagnosis of lung cancer in Portugal.

Variables/categories	Total (n = 61) n (%)	Exclusively assist lung cancer patients (n = 26)	p-value #
**Perception that the referral process impacts on**			
Diagnosis/staging	43 (70.49%)	20 (76.92%)	0.343
Therapy beginning*	35 (58.33%)	15 (60.00%)	0.825
**Factors preventing referral to the specialty**			
Poor referral network*	26 (43.33%)	13 (50.00%)	0.362
Poor communication between services*	36 (60.00%)	17 (65.38%)	0.457
Patients low socioeconomic level	27 (44.26%)	10 (38.46%)	0.432
Geographical region	6 (9.84%)	4 (15.38%)	0.210
**Factors preventing rapid diagnosis**			
Lack of technical resources	40 (65.57%)	18 (69.23%)	0.604
Lack of human resources	33 (54.10%)	15 (57.69%)	0.627
Poor communication between specialties	17 (27.87%)	7 (26.92%)	0.887
**Factors preventing rapid disease staging**			
Lack of technical resources	51 (83.61%)	21 (80.77%)	0.606
Lack of human resources	35 (57.38%)	15 (57.69%)	0.966
Poor planning	10 (16.39%)	4 (15.38%)	0.854
Factors preventing multidisciplinary consultation**			
Available specialty	24 (39.34%)	8 (30.77%)	0.237
Lack of standard electronic platforms	12 (19.67%)	3 (11.54%)	0.168
Low health literacy	4 (6.56%)	4 (15.38%)	***0*.*016***
**Factors preventing therapy beginning**			
Internal authorization	35 (57.38%)	13 (50.00%)	0.315
INFARMED authorization	46 (75.41%)	20 (76.92%)	0.813
Therapy availability	10 (16.39%)	3 (11.54%)	0.377
Treatments costs	10 (16.39%)	4 (15.38%)	0.854
**Biomarkers analysis are requested**			
Upon histopathological diagnosis	53 (88.33%)	24 (92.31%)	0.835
During specialty consultation	4 (6.67%)	1 (3.85%)	0.472
During multidisciplinary consultation	3 (5.00%)	1 (3.85%)	0.732
**Most time-consuming steps of biomarkers analysis**			
Histology/molecular evaluation	38 (62.30%)	18 (69.23%)	0.335
Obtaining the term for analysis	10 (16.39%)	1 (3.85%)	***0*.*023***
Sample outsourcing	20 (32.79%)	9 (34.62%)	0.793
Started therapy before biomarkers results***			
Worsening performance status	40 (65.57%)	17 (65.38%)	0.979
High tumor burden	32 (52.46%)	12 (46.15%)	0.395
Lack of new drugs	3 (4.92%)	1 (3.85%)	0.739
Patients emotional condition	25 (40.98%)	14 (53.85%)	0.078
**Factors that impact on therapy beginning**			
Patients referral	14 (22.95%)	6 (23.08%)	0.984
Diagnosis	18 (29.51%)	8 (30.77%)	0.852
Disease staging	29 (47.54%)	13 (50.00%)	0.740
Biomarkers analysis	32 (52.46%)	14 (53.85%)	0.852
Internal approval	14 (22.95%)	4 (15.38%)	0.226
External approval	24 (39.34%)	10 (38.46%)	0.903
**Perception on potential time optimization during**			
Patients referral	20 (32.79%)	6 (23.08%)	0.164
Diagnosis	25 (40.98%)	11 (42.31%)	0.856
Disease staging	36 (59.02%)	14 (53.85%)	0.479
Biomarkers analysis	40 (65.57%)	18 (69.23%)	0.604
Internal approval	22 (36.07%)	7 (26.92%)	0.200
External approval	26 (42.62%)	12 (46.15%)	0.631

*Total sample: n = 60 physicians that answered this specific question.

**Total sample: n = 57 physicians that answered to have multidisciplinary consultations on their institutions.

***Total sample: n = 54 physicians that started therapy before receiving the results of biomarkers analysis.

# Pearson chi-square test.

The sample was composed by pulmonologists (n = 45; 73.77%) and oncologists (n = 16; 26.23%), having more than 15 years of experience (mean 16.19 years, 95% CI 13.34–19.05), mostly working in public hospitals (90.16%), especially in Lisbon e Vale do Tejo (36.07%) and North (34.43%) regions. Physicians performed a median of 85 (IQR 55–140) diagnosis of lung cancer during the last semester of 2019, with both primary care and hospital urgencies as main patient’s referral pathway. Overall, n = 26 (42.62%) physicians were considered to have their clinical practice exclusively dedicated to lung cancer patients.

Most physicians agree that the referral process has an important impact both on patients’ diagnosis and staging (70.49%) and beginning therapy (58.33%). The main barriers highlighted by the experts to prevent patient’s rapid referral to the specialty include poor communication between services (60.0%), patients low socioeconomic and cultural levels (44.26%) and poor referral network (43.33%), with no differences among the total sample and physicians that only assist lung cancer patients (p>0.05). Among the factors that prevent both rapid diagnosis and disease staging, lack of technical and human resources were the most frequently identified (>50% of physicians). The vast majority of responders (n = 57; 93.44%) stated that their working centers have multidisciplinary team meetings (MDTMs). According to 39.34% (24/61) of physicians, the availability of a particular medical specialty in a given center can impact on MDTMs.

More than half of experts (n = 33; 54.10%) considered that the time for diagnosis/staging influences therapeutic approach. Overall, regarding non-reimbursed drugs, that correspond to a small percentage of patients’ treatment, obtaining INFARMED (National Drugs Authority) authorization or internal (within the hospital) authorization to begin treatment were considered as major barriers (75.41% and 57.38%, respectively), with no differences among the total sample and physicians that only treat lung cancer patients (p>0.05). Most responders (88.33%) affirmed that biomarkers analyses are usually requested upon histopathological diagnosis. Physicians that assist exclusively lung cancer patients significantly reported less delays for obtaining the term for biomarkers analysis (p = 0.023). The majority of experts (n = 54; 88.52%) stated that in some point of their clinical practice they had the need to start therapy before biomarkers results because of patients’ worsening performance status (65.57%), high tumor burden (52.46%) and patients’ emotional condition (40.98%), with no differences compared to physicians assisting solely lung cancer patients. According to the responders, the steps that most impact on treatment delay and could be optimized are disease staging and biomarkers analysis (see Table 2).

**Fig 1:**
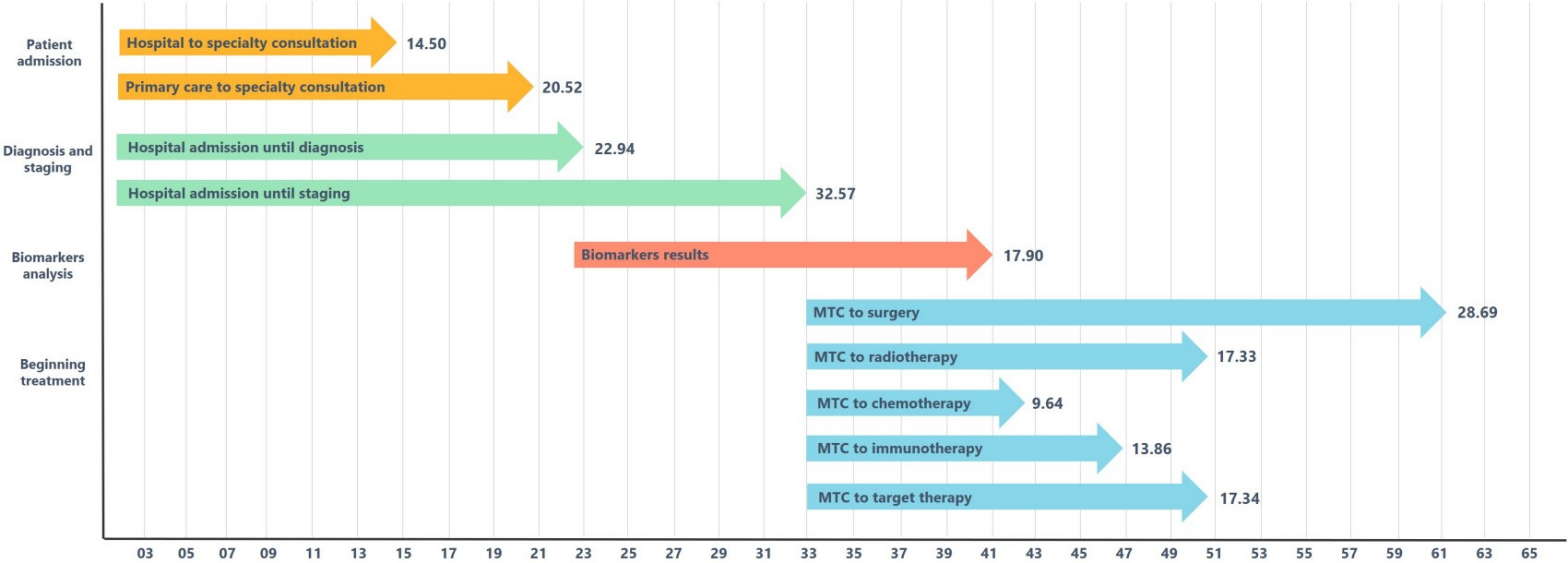
Summary of the main stages and median lag times (days) for lung cancer diagnosis in Portugal (MTC: Multidisciplinary consultation).

Fig 1 summarizes the main stages and lag times perceived by the physicians for the diagnosis of lung cancer in Portugal. Table 3 presents a comparison of these lag times in the perception of the overall sample of physicians and those that exclusively assist lung cancer cases. The median time from primary care or hospital urgency admissions until specialty consultations were of 20.52 days (95% CI 16.70–24.34) and 14.50 days (95% CI 11.33–17.67), respectively. Physicians that exclusively assist lung cancer patients significantly perceived less delays from hospital admission until diagnosis (19.50 days [95% CI 14.92–24.08] vs. 22.94 [95% CI 20.44–25.44]; p = 0.006). The time from multidisciplinary consultation until treatment beginning ranged from 9.64 days (95% CI 8.36–10.91) for chemotherapy until 28.69 days (95% CI 24.55–32.83) for surgery. Physicians that exclusively assist lung cancer patients significantly perceived less delays to start immunotherapy (p = 0.003) or target therapy (p = 0.028). The cumulative lag time from patients’ first admission until beginning of therapy varied from around 42 days (6 weeks) for chemotherapy to around 50 days (7 weeks) for radiotherapy or target therapy, until around 61 days for surgery (almost 9 week) (see Fig 1).

**Table 3:** Time lags to diagnosing lung cancer in Portugal.

Variables/categories	Median time (days) [95% CI]				p-value #
	N	Total	N	Exclusively assist lung cancer patients	
**Time to procedures**					
From primary care until specialty consultation	58	20.52 [16.70–24.34]	24	20.13 [13.82–26.43]	0.727
From hospital admission until specialty consultation	57	14.50 [11.33–17.67]	23	16.23 [10.78–21.68]	0.284
From hospital admission until diagnosis	56	22.94 [20.44–25.44]	21	19.50 [14.92–24.08]	***0*.*006***
From hospital admission until staging	59	32.57 [29.12–36.02]	24	29.75 [23.94–35.56]	0.088
From multidisciplinary consultation until surgery	61	28.69 [24.55–32.83]	26	29.08 [21.67–36.48]	0.970
From multidisciplinary consultation until radiotherapy	61	17.33 [15.05–19.61]	26	16.96 [14.17–19.75]	0.811
From multidisciplinary consultation until chemotherapy	61	9.64 [8.36–10.91]	26	8.34 [7.21–9.48]	0.103
From multidisciplinary consultation until immunotherapy	61	13.86 [11.94–15.84]	26	10.77 [8.77–12.76]	***0*.*003***
From multidisciplinary consultation until target therapy	61	14.34 [12.26–16.43]	26	11.84 [9.34–14.35]	***0*.*028***
Waiting time for biomarkers results	61	17.90 [16.31–19.49]	26	18.04 [15.90–20.18]	0.761
**Physicians’ perception on time optimization**					
How long is possible to optimize disease diagnosis?	57	11.05 [9.61–12.50]	24	8.75 [6.40–11.10]	***0*.*010***
How long is possible to optimize disease staging?	58	12.79 [11.06–14.52]	25	11.48 [8.73–14.23]	0.222
How long is possible to optimize the begin of therapy?	58	13.16 [11.11–15.20]	25	10.92 [8.14–13.70]	0.063

CI: confidence interval.

# Mann-Whitney U-Test.

Professionals working at private hospitals also reported a lower median time from multidisciplinary consultation to the beginning of both surgery, radiotherapy and immunotherapy when compared to those from public hospitals (p<0.001, p = 0.004 and p = 0.003, respectively). According to the expert’s perception, time for diagnosis can be optimized in all steps of the process (diagnosis, disease staging and beginning of therapy) in around 10–15 days. Physicians that treat only lung cancer patients stated that time for diagnosis can be reduced in 8.75 days (95% CI 6.40–11.10) while the overall sample answered a median time of 11.05 days (95% CI 9.61–12.50) (p = 0.010). Responders working at public hospitals believe that diagnosis can be optimized in further 11.58 days (95% CI 10.14–13.01) compared to 5.60 days (95% CI 1.67–12.87) reported by experts from private institutions (p = 0.023)

## Discussion

This study was triggered by the ongoing debate on the need for improve lung cancer diagnosis and reduce the burden of this disease caused by the high rates of morbidity and mortality in Portugal. Through a nationwide survey with physicians that routinely treat lung cancer patients (i.e. oncologists and pulmonologists) from all Portuguese regions, we were able to identify their perception on some barriers for rapid diagnosis, disease staging and treatment beginning that may impact on clinical and economic outcomes.

The interval from signs and symptoms to lung cancer diagnosis and treatment remains longer than recommended in several countries [21–23], as previous demonstrated in scoping reviews (ranging from 6 to 45 days) [5, 17]. Overall, we found a cumulative delay of around 42 days (6 weeks) from patients’ first admission until beginning of chemotherapy, 50 days (7 weeks) for radiotherapy and around 61 days (8.75 weeks) for surgery. In Australia, a retrospective cohort also reported median times from referral to diagnosis, to first treatment and to surgery of 21, 56 and 70 days, respectively [21]. Similarly, in the United States, median times from first presentation to a specialty clinician until first treatment are around 52 days (7–8 weeks), despite American and British guidelines recommending no more than 42 and 62 days of waiting time, respectively [15, 24, 25]. In our study, physicians believe that the time for diagnosis and treatment beginning can be reduced in around 10 days (1.5 weeks) and 13 days (around 2 weeks), respectively, especially according to those assisting different types of tumors (non-exclusively lung cancer) and working in public hospitals.

In Portugal, the National Health Service (*Sistema Nacional de Saúde*) is characterized by an universal coverage with predominant public financing and services provision, although the participation of the private sector has increased in the past years. Public hospitals account for half of institutions in the country (54.4%), specially allocated in the Lisbon and North regions [26]. We found a median of 85 diagnosis of lung cancer per physician just in the last semester of 2019, which could represent around 10300 diagnosis annually. According to the World Health Organization, in 2018, around 5280 new cases of lung cancer were reported in Portugal [3]. The apparently overestimated scenario reported by the interviewed physicians may have occurred due to a tendency to over-report cases, possible duplication of diagnosis, or report of the total number of cases instead of only the new ones. Nevertheless, this scenario enhances the concerns about the prevalence of the disease, especially considering that almost half of the experts exclusively assist lung cancer patients.

The concept of barriers to quality care (such as the receipt of timely and appropriate diagnostic, staging and treatment selection) is used in the context of improving health care management or prevention programs [7]. In our study, clinicians placed greater emphasis on some factors that prevent rapid referral to specialty such as poor communication between services and referral network, and patients low socioeconomic/cultural levels. Socioeconomic deprivation is associated with reduced survival, more early deaths (within 30–90 days) and lower treatment rates. Possible explanations include a failure to present and seek early care and less effective primary services in deprived communities [27, 28]. In the United Kingdom, clinicians consider referrals from primary to secondary care as the most liable to problems leading to delayed cancer diagnosis [29], which was consistent to the results found in our study. In this context, physicians should receive, among others, more timely information from hospitals regarding their patients and have better access to risk assessment tools and diagnostic services [29]. Additionally, healthcare delays occur due to limited resources in workforce and capacity (e.g. technical, human resources) which may be significant higher in public hospitals [20, 30].

Given the increasingly multimodal approach to the diagnosis, staging, and treatment of lung cancer, the multidisciplinary model is a recommended alternative to delivery care [31, 32]. In theory, this model should promote connectivity among providers and collaboration between providers, patients, and family members. This can shorten the length of time before treatment and establish a plan that is tailored to the patient’s needs [33, 34]. However, this is not the current scenario perceived by Portuguese clinicians. Although most hospitals present MDTMs, barriers such as lack of medical specialty in a given center exist. Additionally, oncology care poses considerable health literacy demands on patients who are expected to understand large amounts of information about complex multidisciplinary treatment over lengths of time [35]. Professionals should be able to enhance the usability of health information and services by improving written materials and verbal communication appropriate to each scenario.

According to the experts, obtaining INFARMED or internal authorization for non-reimbursed drugs was rated as the main barriers to begin treatment. While prior authorizations are intended to ensure medical necessity, these additional requirements can add administrative burden and, in some cases, cause significantly delays for patients’ treatment. Communication and collaboration among stakeholders are needed to efficiently delivery care [36, 37].

Although lung cancer is usually classified according to histological criteria, currently, the discovery of multiple molecular mechanisms underlying the development, evolution, and prognosis of these tumors, has support new therapies. The knowledge of some mutations (e.g. EGFR, ALK, KRAS, ROS1) through biomarkers analyses may guide more tailored and effective treatments [38, 39]. However, biomarkers analyses are considered a time-consuming step of patients’ follow-up. Physicians that assist patients with different types of tumors (non-exclusively lung cancer) significantly reported more delays for obtaining the term for biomarkers analysis, which can be associated with hospitals workflow and system-related issues. Additionally, the large waiting time for obtaining biomarkers results (almost 20 days) may contribute to delaying therapy beginning as reported by the majority of Portuguese experts. Patients worsening of performance status, high tumor burden and patients’ emotional condition were rated as major factors for early treatment. A study performed in the United States also demonstrated median time intervals between test ordered by a physician and receipt of tumor specimen at a test center and between test center and the availability of biomarkers results of 11.5 (IQR 8–18.5) and 14 (IQR 10–17) days, respectively. A significant lapse of 83 (IQR 29–147) days prior to patients actually beginning treatment was also reported [40].

Treatment modality can also be a factor associated with longer waiting times and may differ according to each patient and healthcare setting [21]. Physicians exclusively assisting lung cancer patients and those from private hospitals perceived less delays to start immunotherapy, while in public hospitals high waiting times were reported for radiotherapy and surgery. Surgery was the modality with the longest waiting time. This may occur given system-related factors to each type of treatment, that may include different periods for physician appointments (e.g. pulmonologist, thoracic surgeon, cardiologist, radiation), additional procedures (e.g. computed tomography, positron emission tomography, mediastinoscopy, cardiac/pulmonary function testing), and treatment planning [41].

Finally, many delays in lung cancer care are avoidable through optimized clinical management and further efforts from MDTMs and hospital administration. The National Academy of Medicine (Institute of Medicine) proposes that modern health care systems have some aims for quality improvement including safety, effectiveness, patient-centeredness, efficiency, equity, and timeliness [33, 34]. Regardless of the process or resources available in a given health system, care settings cannot improve time to treatment of lung cancer without measuring it [24]. Additionally, established standards and national benchmarks based on the identified factors related to clinical evaluation, staging and treatment delays can help to improve the process of lung cancer diagnosis. Thus, we strongly suggest that future guideline developers in Portugal consider a median time to be set as desirable to start treatment for lung cancer patients.

Our study has some limitations. Non-probabilistic convenience sampling together with the low-moderate adherence rate to the study (67.03%) may carry out a bias in data collection and due to under-representation of subgroups–as more committed responders usually get involved in lung cancer care, as well as exclusive dedicate lung cancer physicians (42.62%). Although this bias is almost unavoidable in cross-sectional studies, our inferences were made grounded on the results obtained with this sample, without further extrapolation to other medical specialties and geographical regions. We also acknowledged the relatively small sample size with limited number of participants from some regions of the country; nevertheless, the proportional distribution among regions is similar to that observed in the country, and the drawbacks that encompass the cross-sectional design. Yet, we were able to portray the perception of physicians that routinely assist lung cancer patients in Portugal. It is possible that the perceived limitations and barriers found in our study are underestimated. The apparent discrepancy between the official number of lung cancer diagnosis in Portugal and the one reported by the interviewed physicians could be influenced by several factors. In cross-sectional surveys, response bias, which includes recall or memory bias, can either enhances or impairs the recall of a memory or alters the content of a reported fact [42, 43]. It is possible that responders considered the total number of diagnosed lung cancer patients in their healthcare center rather than the number of patients they actually treat, which may lead to some overlapping results. However, in the absence of real data on Portuguese lag-times to lung cancer diagnosis, staging and treatment, the perceived times reported by lung cancer physicians are the best evidence available, which brings this topic for additional reflection. Further analysis of patients’ records could help to understand in detail the delays in the process, aiming at the improvement of the pathway. Although the questionnaire was applied in the beginning of 2020 –which could raise concerns about the impact of the pandemic on the clinical activities evaluated in this study–all questions were retrospective regarding the second half of 2019.

## Conclusions

Portuguese physicians believe that is possible to reduce delays in all stages of lung cancer diagnosis in at least one week. However, further efforts from multidisciplinary teams and hospital administration are needed. Additionally, future guideline developers and decision-makers should consider a median time to be set as desirable for beginning lung cancer treatment in Portugal.
